# Prevalence and Imaging Characteristics of Palatine Tonsilloliths Detected by CT in 2,873 Consecutive Patients

**DOI:** 10.1155/2014/940960

**Published:** 2014-10-16

**Authors:** Akira Takahashi, Chieko Sugawara, Takaharu Kudoh, Daisuke Uchida, Tetsuya Tamatani, Hirokazu Nagai, Youji Miyamoto

**Affiliations:** ^1^Department of Oral Surgery, Institute of Health Biosciences, Tokushima University Graduate School, 3-18-15 Kuramoto-cho, Tokushima 770-8504, Japan; ^2^Department of Comprehensive Dentistry, Institute of Health Biosciences, Tokushima University Graduate School, 3-18-15 Kuramoto-cho, Tokushima 770-8504, Japan

## Abstract

*Aim.* Tonsilloliths are calcified structures that develop in tonsillar crypts. They are commonly detected in daily clinical practice. The prevalence of tonsilloliths was 16 to 24% in previous reports, but it is inconsistent with clinical experience. The aim of this study is to clarify the prevalence, number, and size distribution of tonsilloliths using computed tomography (CT) in a relatively large number of patients. *Materials and Methods.* We retrospectively reviewed the scans of 2,873 patients referred for CT examinations with regard to tonsilloliths. *Results.* Palatine tonsilloliths were found in 1,145 out of 2,873 patients (39.9%). The prevalence of tonsilloliths increased with age, and most commonly in patients of ages 50–69. The prevalence in the 30s and younger was statistically lower than in the 40s and older (*P* < 0.05). The number of tonsilloliths per palatine tonsil ranged from one to 18. The size of the tonsilloliths ranged from 1 to 10 mm. For the patients with multiple CT examinations,the number of tonsilloliths increased in 51 (3.9%) and decreased in 84 (6.5%) of the tonsils. *Conclusions.* As palatine tonsilloliths are common conditions, screenings for tonsilloliths during the diagnosis of soft tissue calcifications should be included in routine diagnostic imaging.

## 1. Introduction

Tonsilloliths (tonsillar concretions, tonsillar calculi) are calcified structures that develop in enlarged tonsillar crypts. Most palatine tonsilloliths are usually asymptomatic. Large palatine tonsilloliths can cause recurrent or persistent throat irritations or discomfort, pain, dysphagia, bad taste, odor, otalgia, and foreign body sensation noted on swallowing [1, 2]. Palatine tonsilloliths are also suspected to be a causative factor of orofacial pain or glossopharyngeal neuralgia [3].

Palatine tonsilloliths may be discovered incidentally on routine panoramic radiographs, which are taken during dental treatment. It is important for the clinicians to differentiate palatine tonsilloliths from pathologic calcified structures such as sialoliths of the parotid/submandibular glands. However, the prevalence and imaging characteristics of tonsilloliths in panoramic radiographs are still unclear, and there is no clue for differential diagnosis by panoramic radiography alone. As panoramic radiographs cannot show the exact location of observed opacities and cannot display the concretions when they are located outside the panoramic focal trough, computed tomography (CT) provides more sensitive and accurate 3D information in detecting calcifications including tonsilloliths [2]. Therefore, basic precise information concerning tonsilloliths, which includes true prevalence, age distribution, and imaging characteristics, should be elucidated through CT. Previous studies showed that the prevalence of tonsilloliths was between 16 and 24% [4, 5], but those reports were based on small samples, and it is inconsistent to clinical experiences.

The purpose of this study was to clarify the prevalence of palatine tonsilloliths using a relatively large series of CT examinations, which were taken for the diagnosis of oral and maxillofacial disease regardless of the presence of palatine tonsilloliths.

## 2. Materials and Methods

### 2.1. Patients and CT Technique

We retrospectively reviewed 8,133 CT examinations, which included the scanning of the head and neck region and were taken for the diagnosis of oral and maxillofacial disease between 2004 and 2012. The CT examinations were taken for the diagnosis of tumors, inflammatory lesions, head and neck injuries, congenital diseases, and other miscellaneous conditions and for the preoperative planning of dental implants. After excluding images of insufficient quality due to heavy metal or motion artifacts and discounting patients with extensive defects of the oropharyngeal region due to surgery, 6,466 CT examinations of 2,873 patients (men 1,340 and women 1,533) were used for further analysis. The signs and symptoms within the tonsillar regions were not considered.

CT devices used were Somatom (Siemens, Erlangen, Germany) with single-row or Aquilion (Toshiba, Tokyo, Japan) with 16-row multidetectors. The reconstruction thickness was 1 mm. The scanning plane was parallel to the occlusal plane and/or the inferior border of the mandible to minimize regions with dental metallic artifacts.

### 2.2. Image Analysis and Statistical Analysis

**Figure 1:**
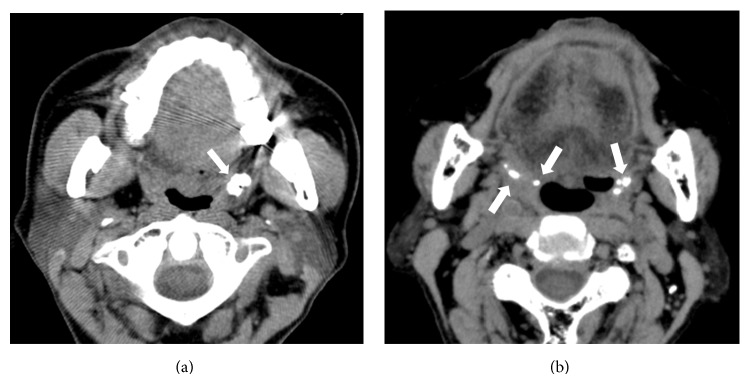
Axial CT images. (a) Single large tonsillolith in the left palatine tonsil (white arrow). (b) Multiple small tonsilloliths involving right and left palatine tonsils (white arrows).

The number and size of the palatine tonsilloliths were counted by displaying the CT images in the monitor with a soft-tissue window by a single, experienced dental radiologist (Akira Takahashi). An example of CT images of palatine tonsilloliths is shown in Figure 1. If the patient had multiple examinations, the first CT examination was used as a reference, and subsequent examinations were used to investigate change with time in the number of palatine tonsilloliths. We used the chi-square and Mann-Whitney *U* tests with a significance level of 5% to determine statistical significance.

This clinical investigation was approved by the Ethics Committee of the Tokushima University Hospital on November 26th, 2012 (number 1580), and informed consent was obtained from all patients before the review of images.

## 3. Results

**Figure 2:**
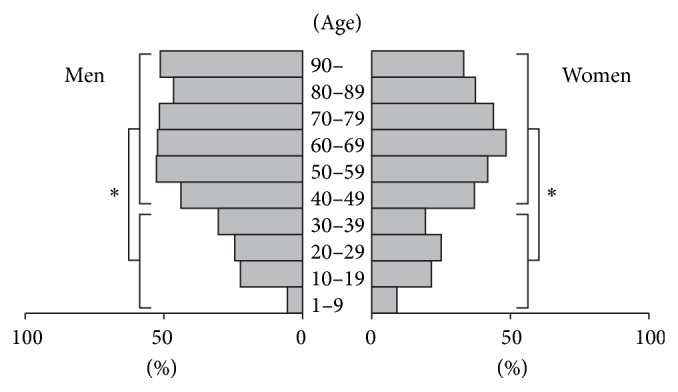
Prevalence of palatine tonsilloliths by sex and age range. The prevalence of palatine tonsilloliths increased with age, ages up to 60–69. The prevalence in patients aged 39 and younger was statistically lower (*P* < 0.05) than those older than 40 in both men and women.

Palatine tonsilloliths were found in 1,145 out of 2,873 patients (39.9%), including 574 men and 571 women. The prevalence was 42.8% in men and 37.2% in women, and it was higher in men (*P* < 0.05, odds ratio = 1.26). The age of patients with palatine tonsilloliths ranged from seven to 98 years, and the mean age was 57.3 ± 17.0 years (57.3 ± 16.2 years for men and 57.3 ± 17.8 years for women), which was significantly older than those without tonsilloliths (mean age of 49.8 ± 20.9 years, 49.0 ± 20.6 years for men, 50.4 ± 21.1 years for women) (Mann-Whitney, *P* < 0.05). The prevalence of palatine tonsilloliths increased with age up to the 60s age range (Figure 2). The prevalence of palatine tonsilloliths in the 30s and younger was statistically lower than in the 40s and older in both men and women (Mann-Whitney, *P* < 0.05). Palatine tonsilloliths were found most commonly in the 50s to 60s in both men and women. In men, its prevalence remained the same above the 50s. In contrast, the prevalence gradually decreased above the 60s in women, although there was no statistical significance between age classes. A unilateral tonsillolith was observed in 709 out of 1,145 patients (61.9%), and bilateral tonsilloliths were observed in 436 patients (38.1%). The most frequent findings were a single unilateral tonsillolith, followed by a single tonsillolith on both sides (bilateral).

A total of 3,141 palatine tonsilloliths were analyzed. Of these, 1,637 (52.1%) and 1,504 (47.9%) were observed in the right and left sides, respectively. There was no significant difference between the left and right sides. The number of tonsilloliths per palatine tonsil ranged from one to 18. A single tonsillolith was found in 865 tonsils (54.7%), two tonsilloliths in 326 tonsils (20.6%), three in 162 (10.2%), four in 91 (5.8%), five in 64 (4.0%), and more than five in 73 (4.6%) (Table 1). The size of palatine tonsilloliths ranged from 1 to 10 mm. Out of 3,141 palatine tonsilloliths, 1,722 (54.8%) were 1 mm in diameter, 904 (28.8%) were 2 mm, 344 (11.0%) were 3 mm, 104 (3.3%) were 4 mm, 50 (1.6%) were 5 mm, and 17 (0.5%) were more than 5 mm (Table 2).

A total of 1,292 palatine tonsils from 646 patients had multiple CT examinations. The interval between examinations ranged from six months to seven years. The change with time in the number of the tonsilloliths per palatine tonsil is summarized in Table 3. The number of tonsilloliths that increased in the range of one to five calculi was 51 tonsils (3.9%), and the number that decreased in the range of one to four calculi was 84 tonsils (6.5%). The number of tonsilloliths that did not change during the observation period was 1,157 tonsils (89.6%).

## 4. Discussion

Tonsilloliths are thought to result from unresolved tonsillitis; infectious agents, such as fungi, bacteria and actinomyces, combined with pus cells serve as an ideal location for stone formation [6]. Large palatine tonsilloliths are rare conditions, and only approximately 50 cases have been reported in the literature [1–3, 6–14]. Tonsilloliths may vary in size and shape, such as round or rod shaped. They may also arise as single or multiple and unilateral or bilateral formations. Pruet and Duplan [1] reported that they can occur at any age, but the occurrence of palatine tonsilloliths in children is unusual. They are most frequently detected in the fourth decade of life. No predilection for gender has been reported [1, 8].

In the present study, we reviewed CT studies from 2,873 patients. This report is the first to clarify the nature of palatine tonsilloliths in a relatively large number of patients. In previous reports [4, 5], palatine tonsilloliths were found in 16–24.6% of patients in sample sizes of 100 to 150 individuals. In our results, palatine tonsilloliths were found in 1,145 (39.8%) patients, which was higher than what was previously reported. In some cases, small tonsilloliths remain undetected due to the partial volume effect caused by the thicker reconstruction thickness (5–10 mm) of CT images in previous reports. We found that palatine tonsilloliths were approximately 1.2-fold prevalent in men than in women, which was a statistically significant result that differed from previous reports [1, 4–6]. The prevalence of tonsilloliths was slightly higher in men older than 50 years (Figure 2). Although the reason is unclear, it is possible that chronic oropharyngeal inflammation persists in elderly men due to the higher rates of smoking and/or poor oral hygiene [15, 16]. The age of the patients with palatine tonsilloliths ranged from seven to 98 years, which means that palatine tonsilloliths can occur in any decade of life. The average age of patients with palatine tonsilloliths at the time of CT examination was 57.3 years, which was consistent with the result of Aspestrand and Kolbenstvedt [4]. Similar to previous reports by Pruet and Duplan [1] and Ram et al. [9], we found palatine tonsilloliths in less than 10% of patients younger than 10, suggesting that palatine tonsilloliths are unusual in children. Previous reports indicate that that palatine tonsilloliths occur most commonly in young adults [1], in the 30s [8] or 40s [12] or randomly in different age ranges [5]. In our study, however, the prevalence of palatine tonsilloliths significantly increased in patients over 40 and was highest in the 50s and 60s. The prevalence was also high in the older age group. Although the reason is unclear, it is possible that the higher prevalence of recurrent oropharyngeal infection in elderly people leads to palatine tonsilloliths. There was an equal side distribution, and a single unilateral palatine tonsillolith was most common, as previously reported [1, 4–7, 9].

The change with time concerning palatine tonsilloliths has not been reported previously. Although palatine tonsilloliths may extrude spontaneously [6], new tonsilloliths usually develop. In the present study, the number of tonsilloliths did not change in 89.6% of the palatine tonsils during six months to seven years of our observation period. Thus, many palatine tonsilloliths were stable for long periods of time. However, new tonsilloliths may develop and spontaneous excretion may occur around 10% of the population.

Palatine tonsilloliths are detected on lateral survey radiographs of the pharynx [17] or panoramic dental radiographs [2, 3, 8–12]. We also occasionally encountered other types of calcified structures that covered the soft-tissue or mandible on plain radiographs. These structures included calcifications of arteries, lymph nodes and salivary glands, phleboliths, an elongated styloid process, a large maxillary tuberosity, a prominent hamulus of the pterygoid process, foreign bodies, bone islands in the mandibular rami, and a displaced tooth [4, 6, 7, 10, 13, 17, 18]. Although CT offers complete and accurate information concerning the location and nature of these types of calcifications [13], they should be differentiated from each other as clearly as possible with plain radiographs to avoid unnecessary radiation exposure.

## 5. Conclusion

Palatine tonsilloliths were common forms of calcification in the soft tissue. Radiologists should be aware that palatine tonsilloliths emerge frequently, and they should be included among the diagnostic possibilities when conventional plain radiographs show soft-tissue calcifications.The prevalence and imaging characteristics of tonsilloliths in panoramic radiographs will be dealt with in the subsequent article.

## Figures and Tables

**Table 1:** Number of tonsilloliths per palatine tonsil.

Number of tonsilloliths	Number of tonsils	(%)
1	865	(54.7)
2	326	(20.6)
3	162	(10.2)
4	91	(5.8)
5	64	(4.0)
>5	73	(4.6)

Total	1581	(100)

**Table 2:** Distribution of the size of palatine tonsilloliths.

Size (mm)	Number of tonsilloliths	(%)
1	1722	(54.8)
2	904	(28.8)
3	344	(11.0)
4	104	(3.3)
5	50	(1.6)
>5	17	(0.5)

Total	3141	(100)

**Table 3:** Change with time in the number of tonsilloliths per palatine tonsil.

Tonsilloliths	Number of tonsils	(%)
Increase	51	(3.9)
No change	1157	(89.6)
Decrease	84	(6.5)

Total	1292	(100)
